# Effects of an electric field on sleep quality and life span mediated by ultraviolet (UV)-A/blue light photoreceptor CRYPTOCHROME in *Drosophila*

**DOI:** 10.1038/s41598-021-99753-4

**Published:** 2021-10-15

**Authors:** Haruhisa Kawasaki, Hideyuki Okano, Takaki Nedachi, Yuzo Nakagawa-Yagi, Akikuni Hara, Norio Ishida

**Affiliations:** 1 Institute for Chronobiology, Foundation for Advancement of International Science (FAIS), 3-24-16 Kasuga, Tsukuba, Ibaraki 305-0821 Japan; 2 Hakuju Institute for Health Science Co., Ltd., 37-5 Tomigaya 1-chome, Shibuya-ku, Tokyo, 151-0063 Japan; 3 Advanced Institute of Innovative Technology, Saitama University, Saitama, Japan; 4 Tsukuba Innovation Arena, National Institute of Advanced Industrial Science and Technology (AIST), 1-1-1 Higashi, Tsukuba, Ibaraki 305-8566 Japan

**Keywords:** Animal behaviour, Behavioural genetics

## Abstract

Although electric fields (EF) exert beneficial effects on animal wound healing, differentiation, cancers and rheumatoid arthritis, the molecular mechanisms of these effects have remained unclear about a half century. Therefore, we aimed to elucidate the molecular mechanisms underlying EF effects in *Drosophila melanogaster* as a genetic animal model. Here we show that the sleep quality of wild type (WT) flies was improved by exposure to a 50-Hz (35 kV/m) constant electric field during the day time, but not during the night time. The effect was undetectable in *cryptochrome* mutant (*cry*^*b*^) flies. Exposure to a 50-Hz electric field under low nutrient conditions elongated the lifespan of male and female WT flies by ~ 18%, but not of several *cry* mutants and *cry* RNAi strains. Metabolome analysis indicated that the adenosine triphosphate (ATP) content was higher in intact WT than *cry* gene mutant strains exposed to an electric field. A putative magnetoreceptor protein and UV-A/blue light photoreceptor, CRYPTOCHROME (CRY) is involved in electric field (EF) receptors in animals. The present findings constitute hitherto unknown genetic evidence of a CRY-based system that is electric field sensitive in animals.

## Introduction

Electric fields (EFs) influence various behaviors, including embryogenesis, wound healing, polarity, differentiation, and motility in plants and animals^1–3^. However, how electrical cues are received and translated into a cellular response in most life systems is poorly understood.

High voltage EFs are effective for treating stiff shoulders, headaches, insomnia and chronic constipation^4^. The Japanese Ministry of Health, Labor and Welfare has approved a device that delivers high-voltage electrical potentials (HELP) to the body. Although EF therapy was discovered ~ 60 years ago, the molecular mechanisms of its benefits have remained unknown^3^, despite investigations involving humans and mice^5^. Therefore *Drosophila* melanogaster was the experimental model in the present study.

Several studies have found that animals and plants detect magnetic fields through CRY proteins. Magnetic fields affect the behavior of cockroaches^6^, flies^7–9^, butterflies^10^, birds^11,12^ and humans^13^. Nerve-specific *cry* expression in *Drosophila* increased magnetic field sensitivity^14^. A specific geographic magnetic field is imprinted on *Drosophila* and is inherited to its progeny^15^. The *cryptochrome (cry*) gene plays a critical role in magnetic detection among diverse species. Qin et al., reported that CRY protein forms complexes with MagR protein in the retina of migratory birds suggesting they may recognize geomagnetics^7^.

A moving electric charge generates a magnetic field, but the fixed device we used in this study did not generate a strong magnetic field in space. The purpose of the present study is to test the hypothesis that an EF exposure of 50 Hz can improve sleep quality and extend the lifetime in WT flies and that *cry* is essential for the electric field receptor system in *Drosophila.*

## Results

### Daytime exposure to an electric field improves sleep quality.

**Figure 1:**
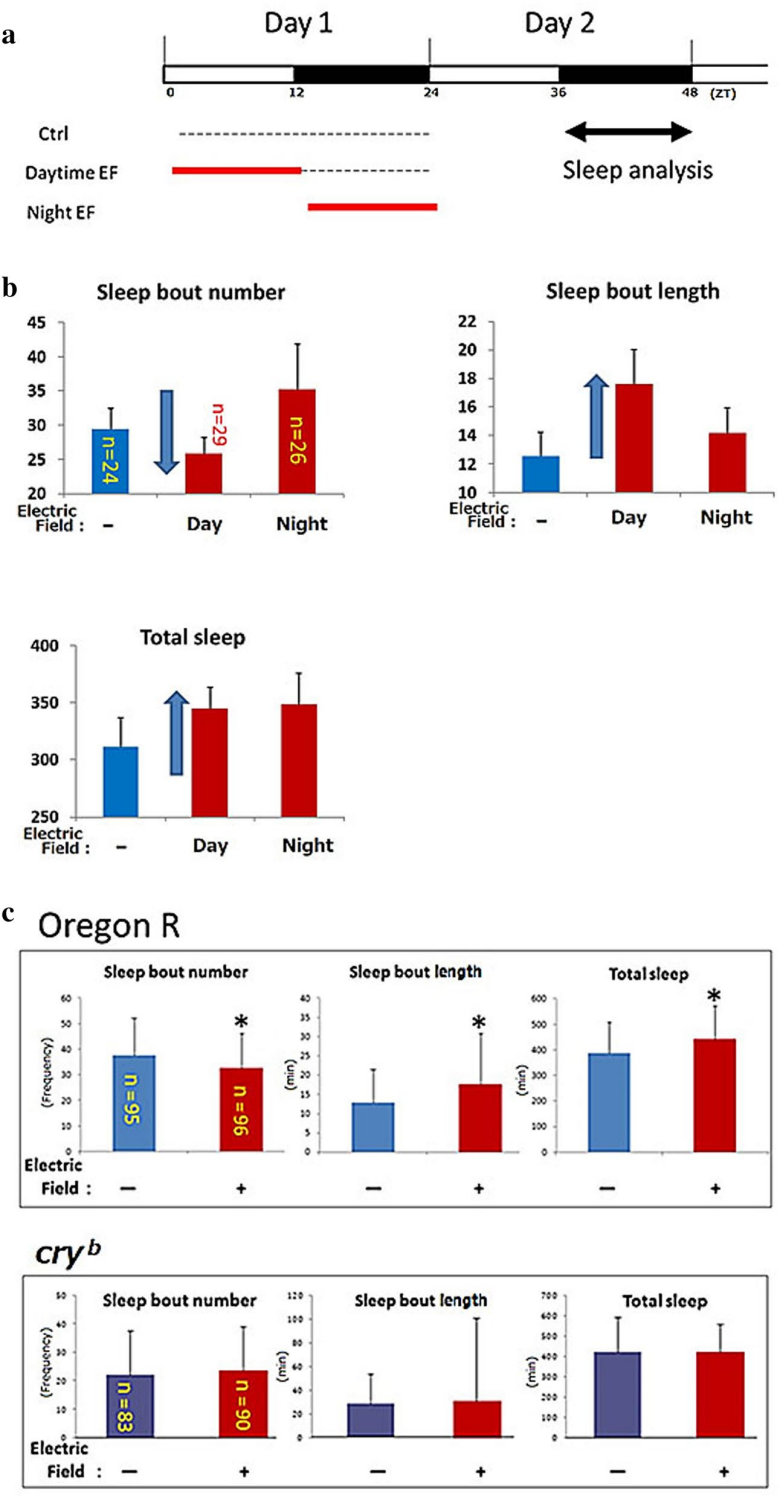

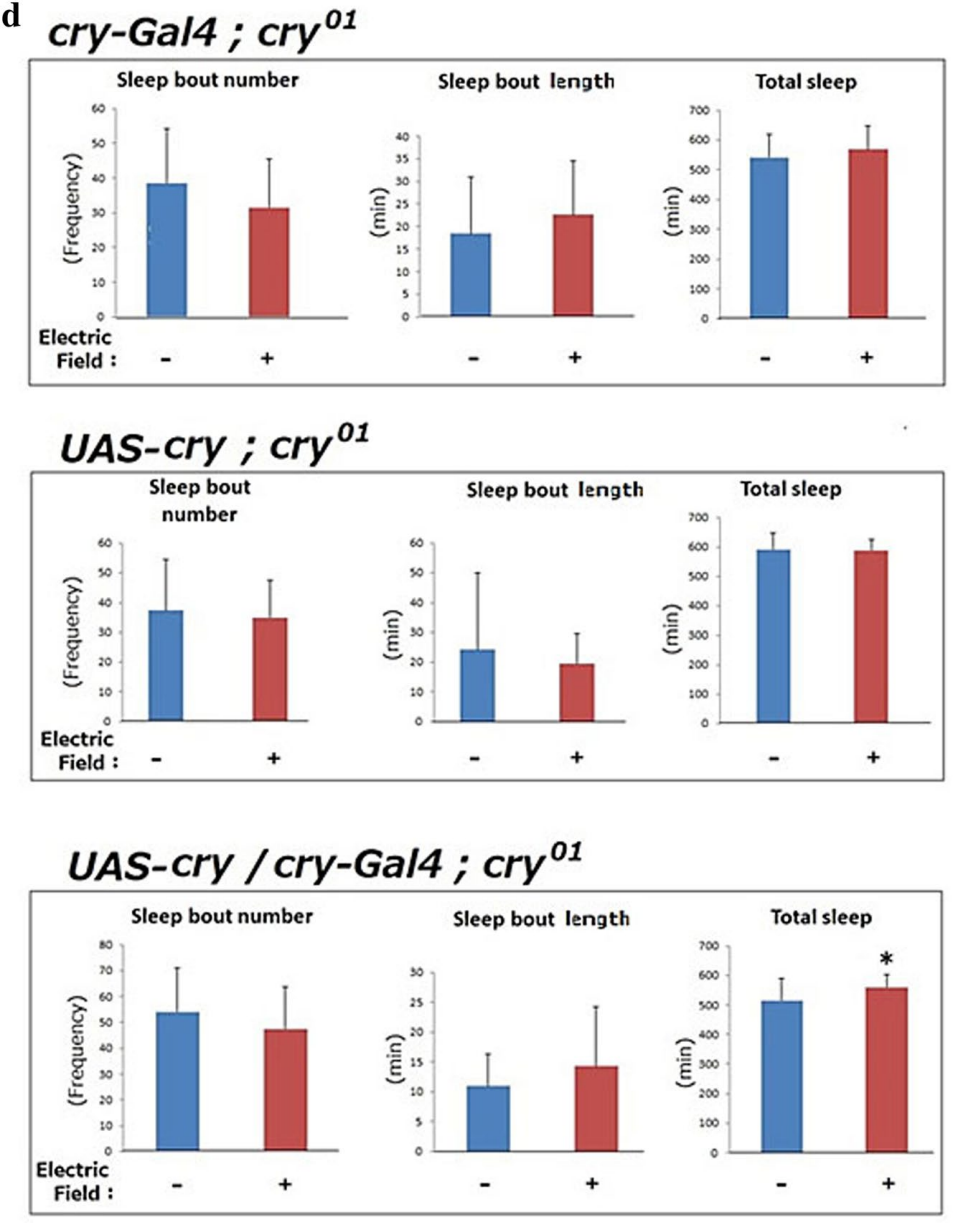
Sleep quality was significantly improved by daytime EF exposure. (**a**) Assessment of EF effects on sleep in WT *Drosophila* (Oregon R). Nighttime sleep was analyzed after daytime EF (DEF) or nighttime EF (NEF) exposure to EF (AC35 kV/m, 50-Hz) for 12 h. (**b**) Comparison of DEF and NEF exposure (shown above) on nighttime sleep between WT *Drosophila* on day 2. Averaged number of sleep bout, averaged length of sleep bout, and total sleep were calculated from locomotor activity data as reported by Ito et al.^25^. Error bars indicate standard deviation. Small number and long length of sleep bout indicates that sleep fragmentation was prevented. Sleep bouts, duration and total sleep were determined in sham control flies (−). DEF exposure decreased sleep bout number, and increased sleep bout length and total sleep time in Oregon-R. There is a possibility that DEF exposure improve sleep quality. (**c**) Effects of daytime EF exposure on nighttime sleep in clock mutant *cry*^*b*^ and WT (Oregon R) flies. Two days after EF exposure, we determined sleep analysis as sum of four independent experiments. Number (left), duration (middle) of sleep bouts, and sleep amount (right) to compare EF exposed flies (+) and sham controlled flies (−). Statistical data by Student’s t-test are expressed as mean ± SD. *represents significant differences (p < 0.05). Daytime EF significantly decreased sleep bout number, and increased bout length and total sleep in WT, but did not significantly affect these in *cry* mutant flies. *EF* electric field, *WT* wild type. (**d**) EF dependent sleep quality improvement in *cry*^*01*^ mutant flies was rescued by *cry* promoter driven CRY expression. These three strains with mutated endogenous *cry* gene (*cry*^*01*^) were used for sleep analysis. Each sleep analysis data of *UAS-cry; cry*^*01*^ (EF, n = 9; sham, n = 8) and *cry-Gal4; cry*^*01*^ (EF, n = 17; sham, n = 19) indicated EF exposure is not affect to the sleep quality improvement. But in the *cry* rescued strain, total sleep amount was significantly increased (p < 0.05, t-test). This data suggests a possibility that EF exposure improved sleep quality of flies through rescued *cry* gene expression (EF, n = 21; sham, n = 21).

We assayed sleep in *Drosophila* to determine the effects of a 50-Hz EF on health. The sleep quality of WT flies exposed to a 12-h EF during the day (ZT 0–12) or night (ZT 13–24) differed (Fig. 1a). A pilot study showed that exposure to EF during the day decreased sleep bout number during the night, but increased the sleep bout length and total sleep in WT flies (Fig. 1b). These results suggest that sleep fragmentation was avoided after daytime EF exposure.

We explored this possibility by evaluating sleep in a large number of WT and *cryptochrome* mutant flies. The results of avoidance of sleep fragmentation as those in the WT were the same (avoidance of sleep fragmentation) in the WT but not in a *cryptochrome* mutant, *cry*^*b*^ (Fig. 1c, lower panels). The differences between sham control flies and EF exposed flies were significant (Fig. 1c, upper panels). We introduced Gal4/UAS system to drive *cry* gene in *cry* mutant flies by using *cry*-driver. Although the parents strains (*cry-Gal4; cry*^*01*^ and *UAS-cry; cry*^*01*^) did not exhibit the sleep improvement effect by EF exposure, *cry* gene driven strain (*cry-Gal4/UAS-cry; cry*^*01*^) partially rescued the sleep improvement by EF exposure (Fig. 1d). We concluded that daytime exposure to a 50-Hz EF improved sleep quality via regulating the *cryptochrome* gene.

### Exposure to EF prolongs average life span under low nutrients

**Figure 2:**
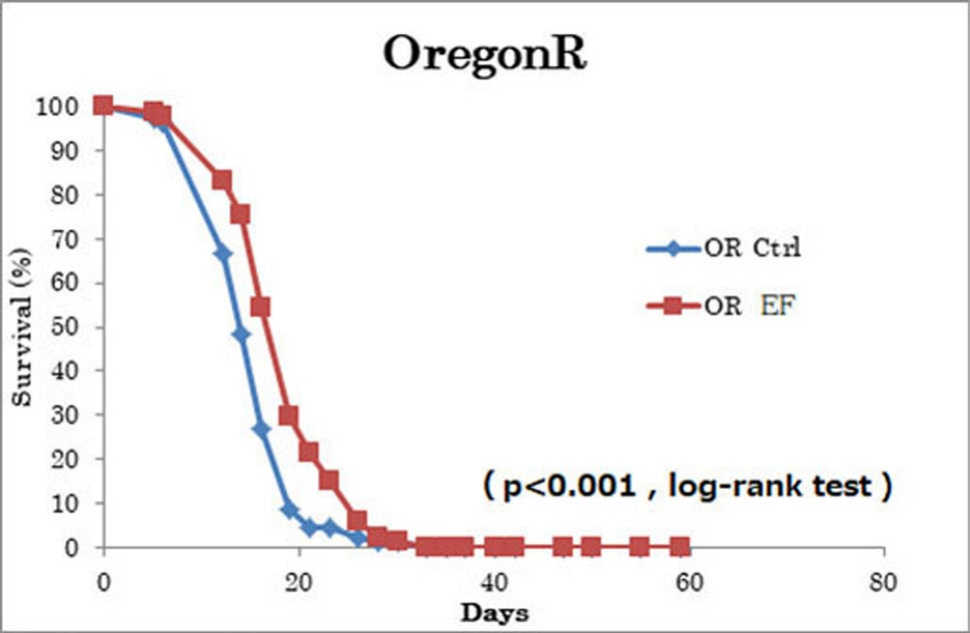
Exposure to EF significantly prolonged average lifespan of WT flies maintained under low nutrient food conditions. Evaluation of senescence status in EF exposed flies (n = 89) versus shame flies (n = 85). Flies under low nutritional status were exposed to 50-Hz EF. This experiment was repeated 3 times. p < 0.001, log-rank test. *EF* electric field, *WT* wild type (Oregon R).

We maintained flies under low nutritional status and then exposed them to EF to determine its effects on lifespan. The average lifespan of WT flies exposed to EF was significantly increased ~ 18% compared with that of sham-treated control flies (Fig. 2). These results showed that exposure to an EF significantly increases the average lifespan of *Drosophila* under low nutritional conditions.

### Exposure EF increases lifespan of *Drosophila* under starvation.

**Figure 3:**
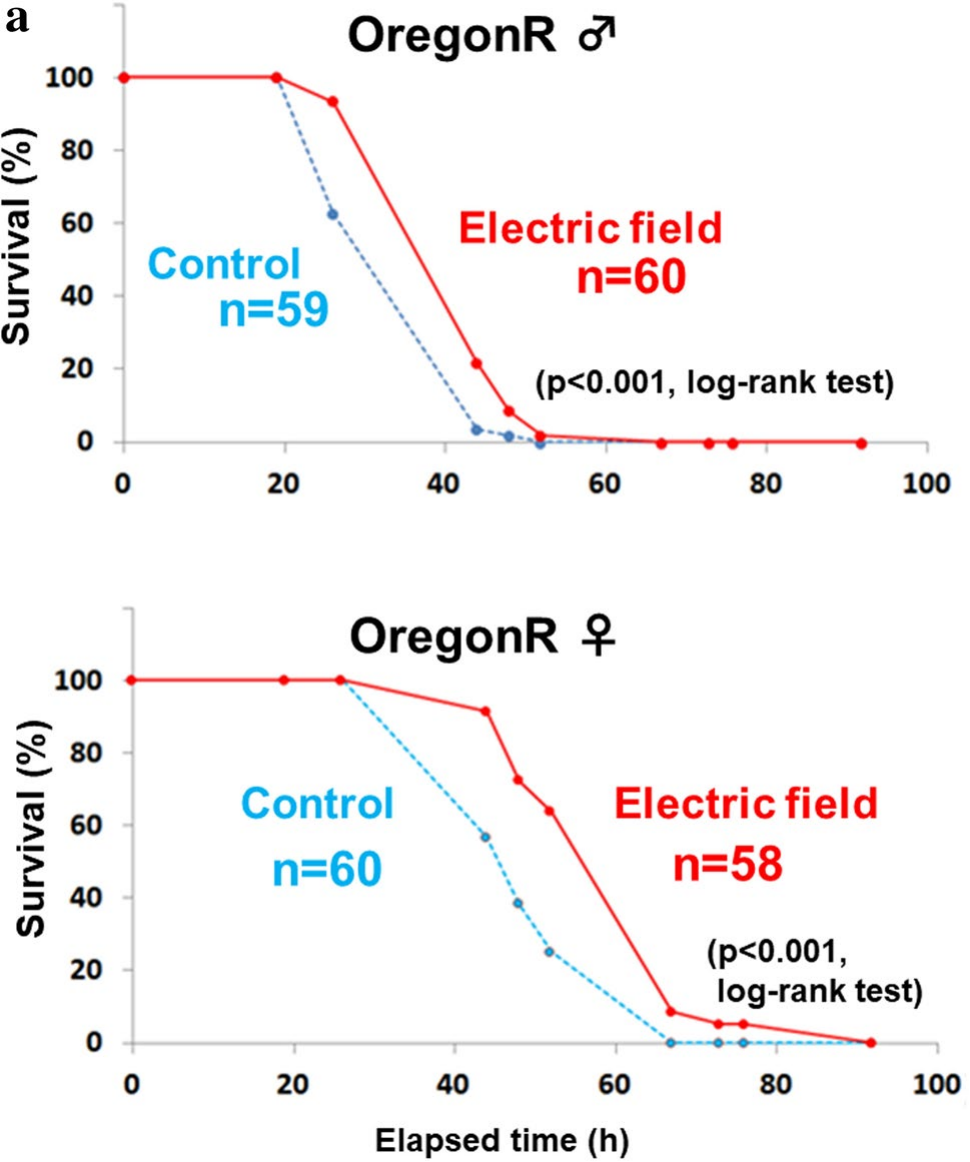

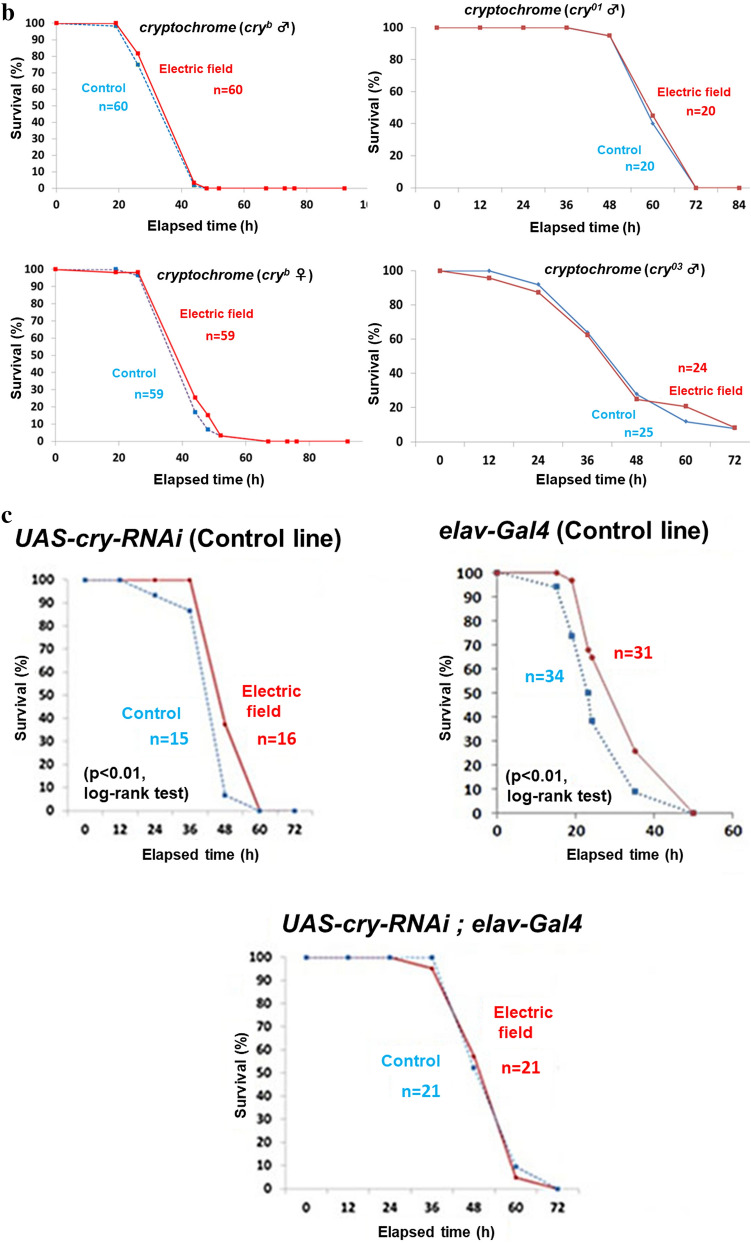

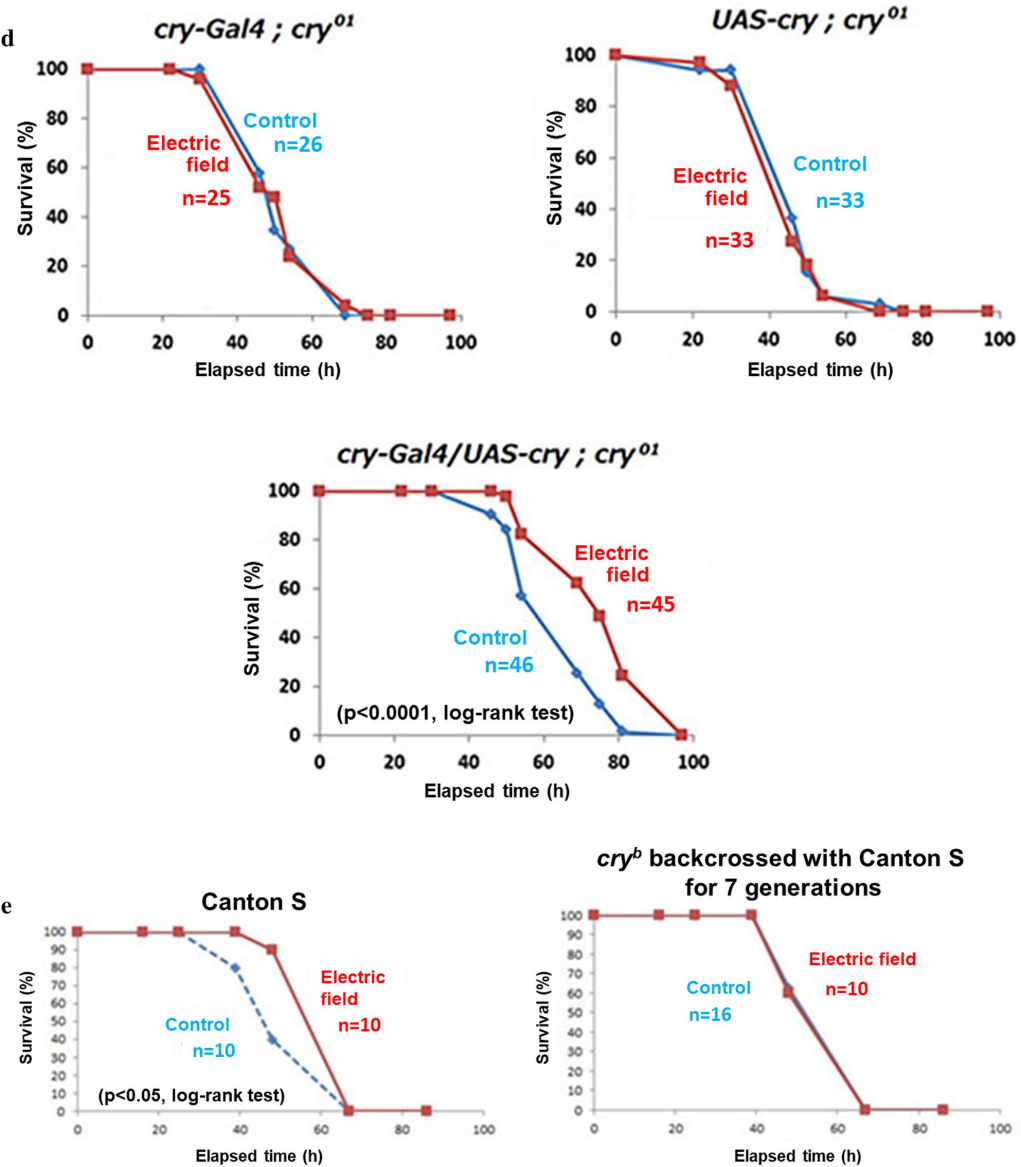
Effects of EF on lifespan of wild type and *cry* gene mutant *Drosophila melanogaster* under starvation. (**a**) The average lifespan of wild type flies (Oregon R) with both of sex was ~ 20% prolonged significantly by 50 Hz EF exposure that started at 2–3 days after eclosion. Starvation was in 1% agarose (that maintained humidity). Upper panel shows the survival curves of male EF exposed flies (n = 60) and male shame controlled flies (n = 59). p < 0.001, log-rank test. Lower panel shows the survival curves of female EF exposed flies (n = 58) and female shame control flies (n = 60). p < 0.001, log-rank test. This experiment was repeated 33 times for male, 3 times for female. (**b**) Lifespan was not significantly elongated in all three types of *cry* mutants. Each panels show survival curves for *cry* mutant flies with EF exposure experiment under starvation. Upper left panel, *cry*^*b*^ male (p = 0.29, log-rank test). Lower left panel, *cry*^*b*^ female (p = 0.23, log-rank test). Upper right panel, *cry*^*01*^ male (p = 0.76, log-rank test). Lower right panel, *cry*^*03*^ male (p = 0.84, log-rank test). This experiment was repeated 26 times for *cry*^*b*^ male, 4 times for *cry*^*b*^ female, 6 times for *cry*^*01*^, 3 times for *cry*^*03*^. (**c**) Survival curves for GAL4/UAS-driven RNAi system for knockdown of *cry* with EF exposure experiment under starvation. Upper panels show the parent lines. Left panel displays *UAS-cry-RNAi* (p < 0.01, log-rank test). EF exposed flies (n = 16) versus shame control flies (n = 15). Right panel displays *elav-Gal4* (p < 0.01, log-rank test). EF exposed flies (n = 31) versus shame control flies (n = 34). Lower panel shows *cry* knock down with using GAL4/UAS-driven RNAi system (p = 0.87, log-rank test). EF exposed flies (n = 21) versus shame flies (n = 21). Exposure to EF significantly increased average lifespan of WT, but not of *cry-*deficient (*cry*^*b*^, *cry*^*01*^, *cry*^*03*^ and *cry* RNAi) lines. In contrast, the average lifespan of control *cry* RNAi parental strains was significantly increased. *EF* electric field, *WT* wild type. This experiment was repeated 5 times for *UAS-cry-RNAi*, 2 times for *elav-Gal4*, 4 times for *UAS-cry-RNAi*; *elav-Gal4*. (**d**) GAL4/UAS-*cry* system rescued cry mutant flies from EF unsusceptibility in survival assay. *UAS-cry* and/or *cry-Gal4* were introduced to endogenous *cry* mutated fly (*cry*^*01*^). Upper panels show survival curves for the parent lines. Upper left panel displays survival curve for *cry-Gal4; cry*^*01*^ (p = 0.87, log-rank test). EF exposed flies (n = 25) versus shame flies (n = 26). Upper right panel displays survival curve for *UAS-cry; cry*^*01*^ (p = 0.57, log-rank test). EF exposed flies (n = 33) versus shame flies (n = 33). Lower panel displays rescue flies, *cry-Gal4/UAS-cry; cry*^*01*^ (p < 0.0001, log-rank test). EF exposed flies (n = 45) versus shame flies (n = 46). This experiment was repeated 2 times for *cry-Gal4; cry*^*01*^, 8 times for *UAS-cry; cry*^*01*^, 2 times for *cry-Gal4/UAS-cry; cry*^*01*^. (**e**) EF exposure did not affect the survival of backcrossed *cry*^*b*^ strains. To compare the effect of EF exposure on homogeneous genetic background flies, *cry*^*b*^ strain backcrossed 7times with wild type (Canton S) was prepared. Right panel displays survival curve for Canton S (p < 0.05, log-rank test). EF exposed flies (n = 10) versus shame control flies (n = 10). Left panel displays the survival curve for *cry*^*b*^ flies backcrossed with Canton S (p = 0.90, log-rank test). EF exposed flies (n = 10) versus shame flies (n = 16). This experiment was repeated 7 times for Canton S, 4 times for *cry*^*b*^, backcrossed with Canton S.

We compared the lifespans of starved male and female WT flies exposed to EF to determine whether the longevity effect would persist. The average lifespan of both sexes were significantly increased by ~ 20% in these flies compared with sham-treated flies (Fig. 3a). These results showed that EF increased the lifespan of WT flies under starvation and in low nutritional conditions.

To confirm that *cryptochrome* is involved in the lifespan elongation mechanism as observed in sleep, we prepared the *cryptochrome* mutants, *cry*^*b*^, *cry*^*01*^, and *cry*^*03*^ (Fig. S1). Interestingly, EF exposure did not alter the lifespan under starvation in any of these mutant lines (Fig. 3b).

We also assessed the EF effect on the lifespan of RNAi mutant flies under starvation (Fig. 3c). Although EF significantly elongated the average lifespan of the parental lines, that of the RNAi *cryptochrome* mutant flies did not differ between sham and EF exposure. When *cry* gene was driven in *cry* mutant fly by using *cry*-driver, EF effect on lifespan elongation was rescued (Fig. 3d). These data indicated that *cryptochrome* is involved in lifespan elongation caused by EF exposure.

Furthermore, we prepared *cry*^*b*^ strain outcrossed 7times with wild type (Canton S) fly to compare lifetime under EF exposed with the homogenous genetic background (Fig. 3e). Predictably, EF exposure elongated lifespan of wild type flies but not that of *cry* mutant with the same background.

### Metabolome analysis showed increased ATP levels in flies

**Figure 4:**
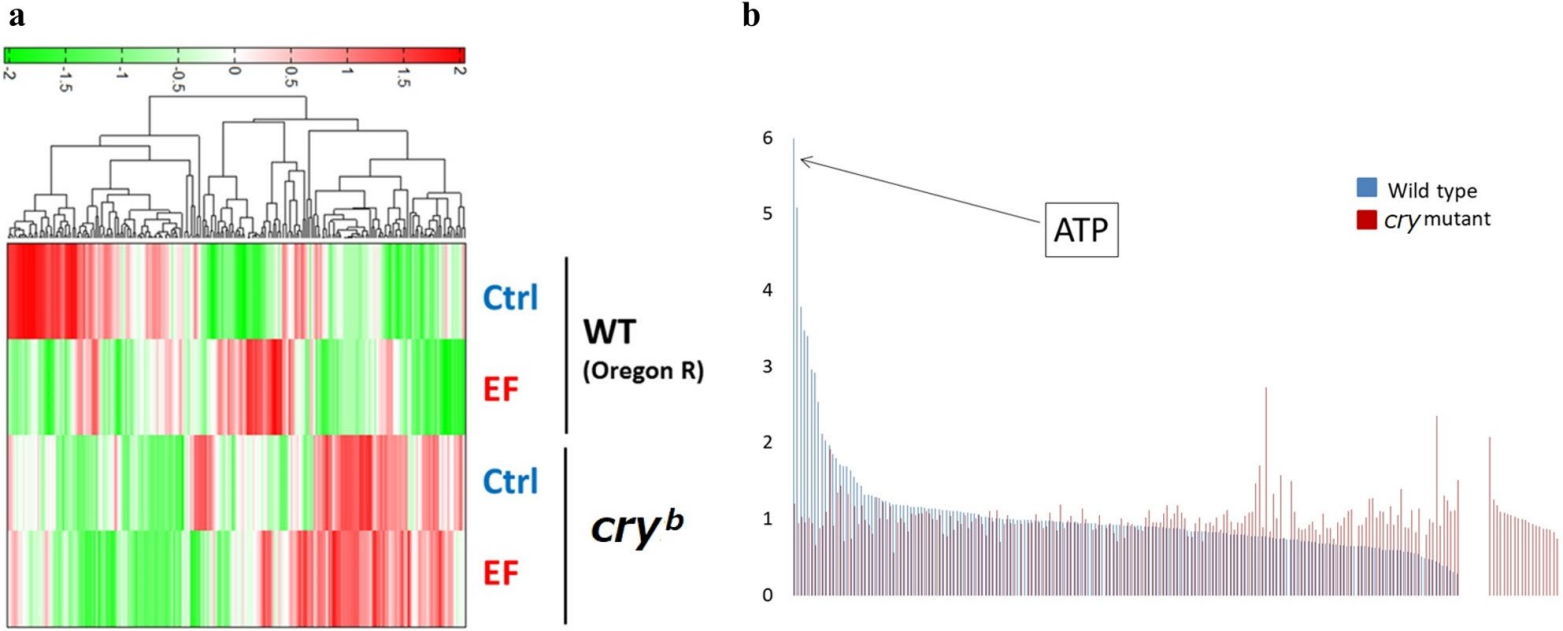
Metabolome analysis after EF exposure. (**a**) Hierarchical cluster analysis of metabolome separation. Heat maps show differences in metabolites between control (Ctrl) and EF exposure. Red and green, high and low expression, respectively. Columns represent different conditions (Ctrl and EF, constant darkness without and with electric field). (**b**) Comparison of upregulated compounds in WT and *cry* mutant flies. Expression of compounds in WT and *cry* mutant is arranged in descending order. ATP is most upregulated by EF. ATP, adenosine triphosphate; *EF* electric field, *WT* wild type.

Although we showed that the *cryptochrome* gene is involved in sleep and the lifespan of flies, the involved metabolic pathways remained unknown. We compared the metabolomes of WT and *cry*^*b*^ mutant flies with or without EF exposure to determine whether EF induces differences in metabolites. Figure 4a shows many differences between control and EF-exposed flies. The difference between Oregon R and *cry*^*b*^ mutant flies might be explained by the genetic backgrounds of the parent flies.

Figure 4b shows an increased ratio of metabolites in EF- compared with sham-treated flies. The graph is arranged in decreased order for wild type flies (blue) and *cry*^*b*^ mutant flies (red). The difference between control and EF-exposed WT was remarkably compared with the *cry*^*b*^ mutant. Table 1 summarizes the data. The abundance of ATP was 5.99-fold higher in EF- than sham-treated flies, but that in *cry*^*b*^ mutant flies was ~ 1.2-fold higher (Fig. 4b, Table 1). To confirm metabolome data, ATP contents after EF exposure was measured by using the luciferase kit (Supplemental Fig. 2). These data indicated that EF exposure significantly increased ATP level and several nucleic acid metabolism in whole fly.

**Table 1:** Top 17 compounds upregulated in WT fly to *cry* mutant fly by EF exposure.

		Wild type	*cry*^*b*^
1	ATP	5.99589	1.20585
2	UDP	5.09161	0.94851
3	GTP	3.79169	1.02959
4	Trehalose 6-phosphate	3.48234	0.95581
5	ITP	3.40563	1.01659
6	Asp	2.97017	0.94291
7	O-Phosphoserine	2.91937	0.65825
8	CDP	2.53999	0.88619
9	PRPP	2.11901	0.91622
10	ADP	2.0375	1.09812
11	Myristoleic acid	1.97591	1.91467
12	UDP-glucose	1.85463	0.91975
	UDP-galactose		
13	3-Phosphoglyceric acid	1.80111	1.35414
14	Fructose 1,6-diphosphate	1.71542	1.43318
15	Threonic acid	1.69059	0.7116
16	Dihydroxyacetone phosphate	1.69036	1.32744
17	Glutathione (GSSG)_divalent	1.64224	0.74731

Abundance of ATP is much more ca. 5.0-fold higher in WT than *cry* mutant.

## Discussion

Exposure to 50 Hz of EF (35 kV/m, constant) during the daytime improved sleep quality in WT flies, whereas nighttime exposure did not. Interestingly, this effect did not observed in *cry*^*b*^ mutant flies. The fact that the number of sleep bouts decreased but the sleep bout length and total sleep time increased with daytime EF exposure suggests that sleep fragmentation was avoided and the duration of each bout of sleep was increased. This phenomenon is considered to be good marker of sleep quality and health in medical science. Exposure to 50 Hz EF at night did not exert such effects. As one of the possibilities, the difference might be explained by the different sensitivity of CRY during day and night. Though cry mRNA expression oscillates with the circadian rhythms, CRY protein does not cycle and simply accumulated in darkness. The protein might be oscillating in LD for light induce CRY degradation. One of possibility is that CRY may be stabilized by EF exposure similarly with magnetic field^8^. Further research is needed to clarify such possibility.

We also found that wild type fly placed in 50 Hz Electric Field elongated average lifespan about 18%. Such lifespan elongation effect was not observed by using several lines including *cry* gene mutant strains and *cry* RNAi strain. CRYPTOCHROMEs (CRYs) are structurally related to ultraviolet (UV)/blue-sensitive DNA repair enzymes called photolyases but lack the ability to repair pyrimidine dimers generated by UV exposure. The *cry* gene was originally identified as a blue light photoreceptor in plants^16,17^. The role of CRY in *Drosophila* is cell-autonomous synchronization and the entrainment of circadian clocks^18^. However, CRY plays a key role in magnetic field detection (8 ~ 14). Here we show that the putative magnetoreceptor protein and UV-A/blue light photoreceptor, CRY is deeply involved in EF receptor in animals. CRY1 and CRY2 have circadian clock functions and may detect magnetic fields in various animals^19^.

Metabolome comparisons between intact WT and *cry* gene mutant strains after exposure to EF indicated that ATP was 5.99 fold more abundant in the WT. Daytime exposure to a 50 Hz EF might activate cellular activities or motility in flies because ATP is the "principal energy currency" in metabolism and the most versatile small molecular regulator of cellular activities^20^. ATP is produced in mitochondria. Interestingly, Alex et al. reported that RNAi of *cry* gene brings degenerated mitochondria in fly heart by coherence microscopy observation^21^. The paper suggests that cry might be involved in mitochondria development and function in *Drosophila*. ATP is produced in the mitochondrial electron transport chain and EF exposure may be involved in the supplier for electron in this system. Quantum biological analysis of electron donor/acceptor might be required for further explanation.

We recognize that this work for EF is different from magnetic field because the magnetic field produced from our device is very weak (Approximately 0.0007 Gauss) to the level on earth (Geomagnetism is 0.5 Gauss). The present findings provide the first genetic evidence of a CRY-based EF sensitive system in animals.

## Methods

### Flies

The *Drosophila* strains Oregon R, *cry*^*b*^, *cry*^*01*^, *cry*^*03*^ a gift from Dr.Paul Hardin, Texas A&M), *w*^*1118*^*; P{GD738}v7239* from the Vienna *Drosophila* Resource Center (VDRC, www.vdrc.at) and *elav-Gal4* were maintained as described^22,23^. The transgenic strain *w*^*1118*^*; P{GD738}v7239* contains upstream regulatory sequences (UAS) to drive the RNAi for the *cry* gene. *UAS-cry; cry*^*01*^ and *cry-Gal4; cry*^*01*^ are gifts from Takashi Yoshii Ph.D. (Okayama University).

### Electric field exposure

We created a uniform EF by transforming a 50-Hz alternating current at 35 kV/m using a Healthtron HEF-P3500 (Hakuju Institute for Health Science Co., Ltd., Tokyo, Japan) to deliver EF. Flies and medium were placed in vials or tubes and placed 3–5 cm apart between the electrodes of the Healthtron for EF exposure.

### Assays of sleep behavior

Male flies were individually placed in glass tubes (inner diameter, 3 mm) containing 5% sucrose and 2% agarose and exposed to EF. The tubes were then transferred to a Drosophila Activity Monitoring (DAM) system (TriKinetics Inc., Waltham, MA, USA) and locomotor activity was measured in 1-min bins. Sleep was defined as > 5 min of consolidated inactivity^24,25^.

### Metabolome analysis

Two days after eclosion, male Oregon R or *cry*^*b*^ flies were exposed to EF for 48 h at DD in starved condition, then whole body flies were stored (30–40 mg batches) frozen in liquid nitrogen. Metabolites were extracted and metabolomes were measured at Human Metabolome Technologies Inc. (HMT, Tsuruoka, Japan. For CE-TOFMS analysis, 1500 μL of 50% acetonitrile (v/v) was added to *Drosophila* samples and blended under cooling using a BMS-M10N21 homogenizer (Bio Medical Science Inc., Tokyo, Japan) at 1500 rpm, 120 s × 5 times). Homogenates were separated by centrifugation at 2300×*g* for 5 min at 4 °C and the upper aqueous layer was centrifugally passed through Millipore 5 kDa cut-of filter (Ultrafree MC-PLHCC, HMT; Millipore Sigma Co., Ltd., Burlington, MA, USA) at 9100×*g* for 120 min at 4 °C to remove macromolecules. The filtrate was then concentrated by centrifugation and reconstituted in 100 μL of Milli-Q water before CE-TOFMS analysis.

Cationic metabolites were analyzed using a fused silica capillary (50 μm i.d. × 80 cm), with the cation electrophoresis buffer H3301-1001 (Human Metabolome Technologies) as the electrolyte. Samples were injected at a pressure of 50 mbar for 10 s, and the applied voltage was 27 kV. Samples were assessed by electrospray ionization-mass spectrometry (ESI–MS) in the positive ion mode, at a capillary voltage of 4000 V. Spectra were obtained between m/z 50 and 1000.

Anionic metabolites were analyzed using a fused silica capillary (50 μm i.d. × 80 cm), with the anionic electrophoresis buffer H3302-1021 (Human Metabolome Technologies) as the electrolyte. Samples were injected at a pressure of 50 mbar for 25 s, and the applied voltage was 30 kV. Samples were assessed by ESI–MS in the negativne ion mode, and the capillary voltage was 3500 V. Spectra were obtained between m/z 50 and 1000.

### ATP contents

ATP extraction was carried out by using Tissue ATP assay kit (TOYO B-Net Co., Ltd., Tokyo, Japan). 150 individuals of male fly whole bodies were gathered and homogenized in ice-cold H buffer [0.25 M sucrose, 10 mM HEPES (pH7.4)] and centrifuged (1000×*g*, 4 °C, 10 min). 1 mL of supernatant was mixed with 7 mL of H buffer and vortex. 100 μL of the mixture was added to 100 μL of ATP extract buffer. After mixed gently, the samples were incubated 30 min in room temperature. Then, ATP amounts were measured by using Wallac 1420 ARVOsx microplate reader (Perkin Elmer Co., Ltd., MA, USA).

### Life span measurements

Lifespan under low nutrient conditions (low nutrient resistance) was assessed by placing male and female flies (typically, 30 individuals each) in vials containing 5 mL of 1% agarose and 5% glucose at 25 °C under LD 12:12 cycles. Dead flies were counted every 2–3 days. Lifespan under starvation in 1% agarose was similarly assayed. Statistical data are expressed as means ± SD. Differences in percent survival was statistically evaluated using log-rank test. Each survival assay was carried out more than two times.

## Supplementary Information

Supplementary Information.
