# Supplementary material for: Effects of an electric field on sleep quality and life span mediated by ultraviolet (UV)-A/blue light photoreceptor CRYPTOCHROME in Drosophila
Source: Sci Rep. 2021 Oct 15;11:20543. doi: 10.1038/s41598-021-99753-4 (PMC8519966; doi:10.1038/s41598-021-99753-4)
Supplement: Supplementary file 1 — Supplementary Information. [file 41598_2021_99753_MOESM1_ESM.pdf]

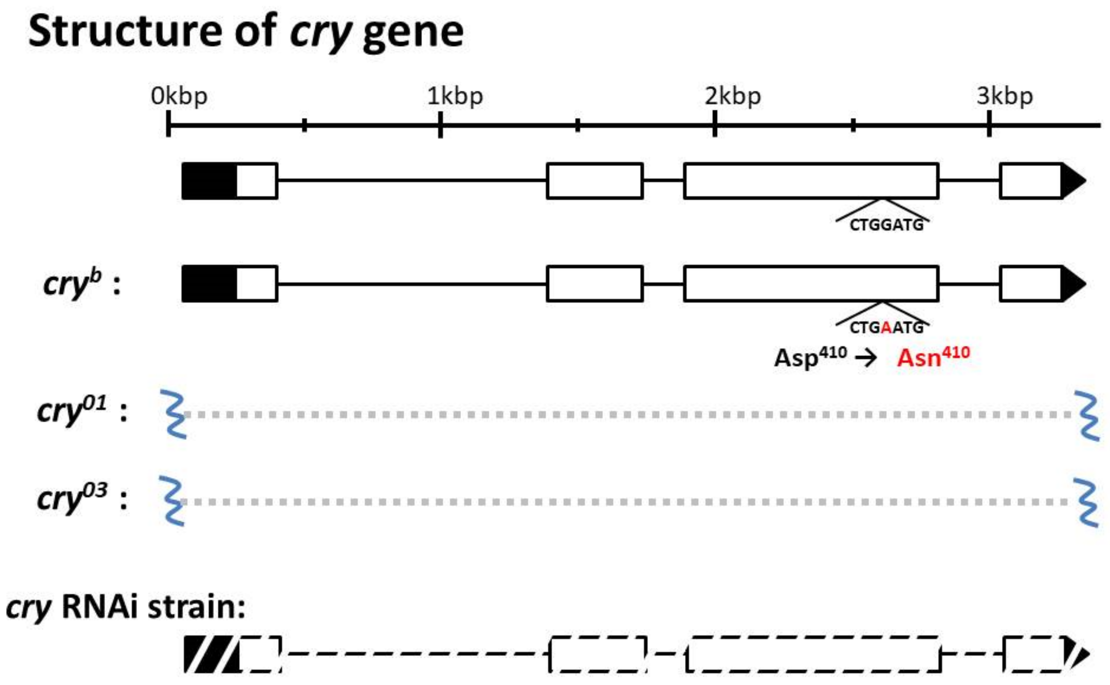

Supplemental figure 1.

Schematic diagram of *cry* gene structure and mutants used in the investigation.

Black squire indicates 5'-UTR (0-187bps), white squires indicate exons (188-328bps, 1330-1673bps, 1830-2749bps, 2986-3208bps respectively). Black triangle indicates 3'-UTR (3209-3288bps). Solid horizontal lines indicate introns. A guanine nucleotide in *cry*^{b} mutant was substituted into adenine at 2571 bps, so that Asp^{410} would be changed to Asn^{410} when it was

translated. *cry*^{01} and *cry*^{03} completely lost *cry* gene because of deletion mutation. *cry* RNAi strain suppress endogenous *cry* gene expression by crossed with elav-gal4 strain.

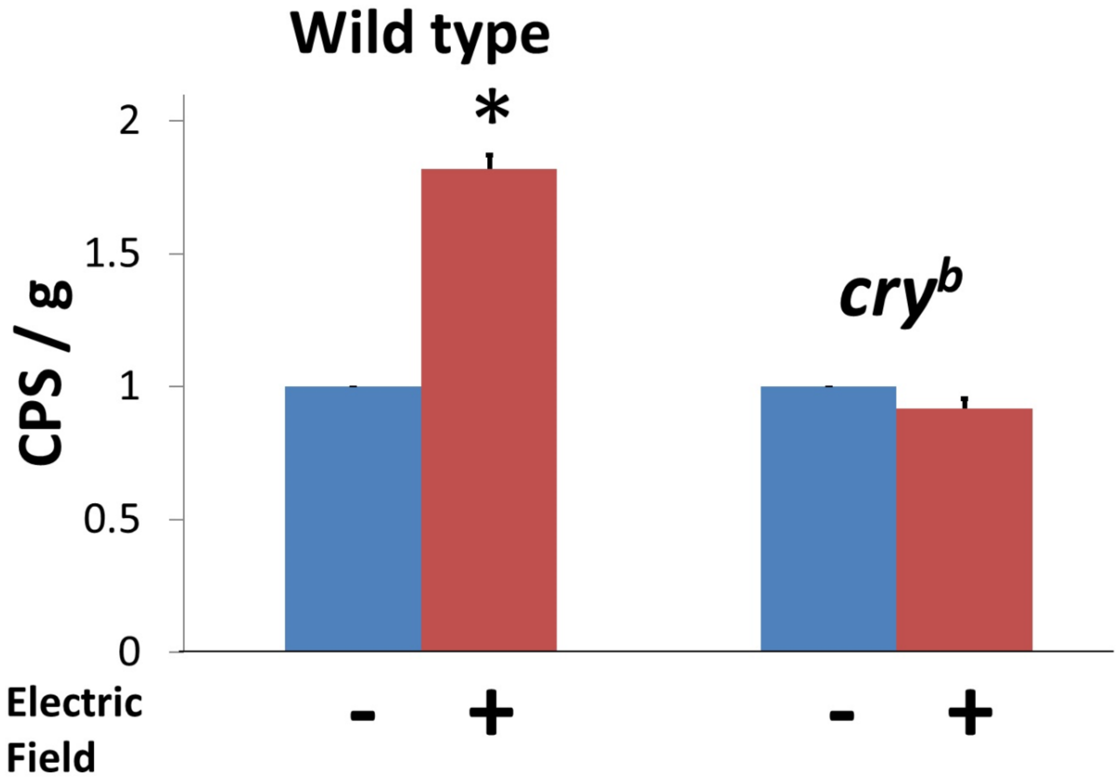

Supplemental figure 2.

Elevation of ATP after EF exposure.

ATP amount was measured by using whole flies after 24hr EF exposure. Though EF exposure increased ATP amount in wild type flies ($p<0.05$, t-test), *cry* mutant flies did not increased. 'CPS/g' indicates 'counter per second / gram of protein'. *cry*^{b}; *cry baby* mutant fly.
